# Pregnancy and patient safety: an overlooked dimension of medical practice

**DOI:** 10.1097/MS9.0000000000004987

**Published:** 2026-04-20

**Authors:** Syeda Nashrah Ayaz, Sabreena Gul, Hafiz Muhammad Faiq Khan, Abedin Samadi, Ahmed Asad Raza

**Affiliations:** a Department of Medicine, Jinnah Sindh Medical University (SMC), Karachi, Pakistan; b Department of Medicine, Kabul University of Medical Sciences Abu Ali Sina, Kabul, Afghanistan

Many physicians experience pregnancy during medical training, yet it is often framed as a personal decision rather than an occupational health and patient safety concern. Pregnancy in medicine poses a serious concern, even from the point of view of the safety of the patients. The maternal and fetal health, along with the clinical and patient well-being, are greatly endangered by the expectation of physicians working during rigorous, risk-laden, time-barred, and physically taxing shifts. Hence, this is a moral and safety problem that needs to be addressed.

Pregnancy represents hidden risks in clinical practice, where fatigue, stress, and medical complications can impair the performance of physicians in delivering patient care. The literature uniformly establishes that overwork and burnout enhance the likelihood of medical errors and expose how impaired performance threatens patient safety[1]. Pregnancy may amplify fatigue, sleep disruption, and physical strain, which can impair attention and decision-making; given the established link between physician fatigue and medical errors, inadequate pregnancy accommodations may indirectly compromise patient safety^[^1,2^]^. This issue echoes duty-hour limits: if reducing trainee work hours safeguards patients, pregnancy accommodation must be considered through the same lens of safety. Evidence signals the burden of poor parental leave policies, characterized by vagueness, uneven application, and stigma, that tend to dissuade doctors from taking necessary time off[3]. Secondarily, occupational hazards such as night work, long working hours, and heavy physical workloads are related to miscarriage, preterm birth, and other pregnancy complications and endanger physician and patient safety[4]. Together, these findings emphasize that pregnancy accommodation is not only a question of equity but also a crucial protection for reliable patient care.

Parental leave and pregnancy accommodations in surgical training remain highly inconsistent across institutions and countries. Pregnancy policies for medical trainees vary widely across healthcare systems worldwide. While some countries provide structured parental leave and duty-hour protections, many settings lack standardized policies, leaving accommodations dependent on institutional discretion[5]. Parental leave policies during North American surgical residency have historically lacked clarity and enforcement, with decisions often left to individual supervisors, creating inequity and variable support for pregnant trainees[3]. The trainee surgeons face elevated obstetric complications, pervasive negative stigma, and insufficient duty modifications, although women overwhelmingly supported accommodations such as reduced call or standing time. By contrast, other high-risk professions (e.g., aviation and military) mandate structured accommodations to safeguard health, underscoring the gap in medicine, where unclear policies contribute to unsafe scheduling, delayed care seeking by pregnant physicians, and higher female attrition.

There are many physical and emotional costs associated with being a pregnant resident, as increased workload causes preterm labor, fatigue, increased contractions, bleeding, poor weight gain, and musculoskeletal pain[6]. Moreover, working in overnight shifts has a higher impact on overall health by disrupting the circadian rhythm and hormonal balance of the body, which causes implantation and fetal growth problems[4]. Residents working >55 hours per week are at an elevated risk of complications like placenta previa in the first two trimesters and preterm premature rupture of membranes in the 3rd trimester[7]. Furthermore, residents with profound devotion are afraid of falling behind and face pressure to succeed. One story told by a resident that she had no choice but to conceal her pregnancy and serve as a workhorse for the residents and nursing staff because of pressure[6]. In addition to that, there is a double burden risk of working on patients when the doctors are not feeling well enough to treat them.

Some healthcare systems have implemented mitigation strategies such as structured parental leave, flexible scheduling, and reduced overnight call requirements during pregnancy to support physician well-being and patient safety; however, implementation remains inconsistent across institutions[5].

It is high time we recognize the challenges faced by pregnant residents working during the day and night for humanity due to increased workload or lack of staff. Residents working 55 hours sacrifice their sleep, diet, and health in this crucial process, which can lead to immense complications[7]. To accommodate residents, appropriate reforms should be made. In order to prevent such individuals from compromising their health, the reform should be implemented by accreditation bodies (ACGME, specialty boards, and health ministries), converting the optional benefit protocols to required safety protocols out of goodwill. Pregnant employees should not be required to perform the 24-hour call shift. In this manner, they will be able to do better work and have more time for self-care[7]. In addition to that, pregnant doctors should be compensated with less workload, proper maternity leave, and equal pay check like the workers who are not pregnant without any external pressure applied by the administration or staff. Similar to how wearing seat belts while driving and working certain duty hours used to be controversial before, doctors’ pregnancy and its protocol should now be treated with the same seriousness. In conclusion, pregnancy in medicine is a fundamental problem tied to multiple factors, but primarily referring to patient safety. It is essential to enable safe healthcare and outcomes by safeguarding pregnant physicians. This assumption, and during inaction, poses a risk not only to one but to both, namely, the mother and the child, as well as patients.

To ensure methodological transparency and ethical compliance in manuscript preparation, we adhered to the recently developed TITAN (Transparency in the Reporting of Artificial Intelligence) 2025 guidelines, which outline standards for responsible AI use in biomedical writing and publishing[8].

## Data Availability

No datasets were generated or analyzed during the current study.
